# Generation of a Deep Mouse Brain Spectral Library for Transmembrane Proteome Profiling in Mental Disease Models

**DOI:** 10.1016/j.mcpro.2024.100777

**Published:** 2024-04-25

**Authors:** Shanshan Li, Huoqing Luo, Pan Tang, Cuiping Tian, Ji Hu, Haojie Lu, Wenqing Shui

**Affiliations:** 1 Institutes of Biomedical Sciences and Department of Chemistry, Fudan University, Shanghai, China; 2 iHuman Institute, ShanghaiTech University, Shanghai, China; 3 School of Life Science and Technology, ShanghaiTech University, Shanghai, China; 4 University of Chinese Academy of Sciences, Beijing, China; 5 Department of Anesthesiology & Perioperative Medicine, Xijing Hospital, Fourth Military Medical University, Xi’an, China; 6 CAS Center for Excellence in Brain Science and Intelligence Technology, Chinese Academy of Sciences, Shanghai, China

**Keywords:** transmembrane proteins, G protein-coupled receptors, mental diseases, spectral library, data-independent acquisition, DIA analysis workflows

## Abstract

Transmembrane (TM) proteins constitute over 30% of the mammalian proteome and play essential roles in mediating cell-cell communication, synaptic transmission, and plasticity in the central nervous system. Many of these proteins, especially the G protein–coupled receptors (GPCRs), are validated or candidate drug targets for therapeutic development for mental diseases, yet their expression profiles are underrepresented in most global proteomic studies. Herein, we establish a brain TM protein-enriched spectral library based on 136 data-dependent acquisition runs acquired from various brain regions of both naïve mice and mental disease models. This spectral library comprises 3043 TM proteins including 171 GPCRs, 231 ion channels, and 598 transporters. Leveraging this library, we analyzed the data-independent acquisition data from different brain regions of two mouse models exhibiting depression- or anxiety-like behaviors. By integrating multiple informatics workflows and library sources, our study significantly expanded the mental stress-perturbed TM proteome landscape, from which a new GPCR regulator of depression was verified by *in vivo* pharmacological testing. In summary, we provide a high-quality mouse brain TM protein spectral library to largely increase the TM proteome coverage in specific brain regions, which would catalyze the discovery of new potential drug targets for the treatment of mental disorders.

## Highlights

• A deep mouse brain spectral library including 3043 transmembrane proteins.
• Library-based DIA data analysis integrating three complementary workflows.
• Differentially regulated transmembrane proteins found in two mental diseaes.
• Discovery of a new potential GPCR regulator of depressive-like behaviors.

## Graphical Abstract

**Figure:**
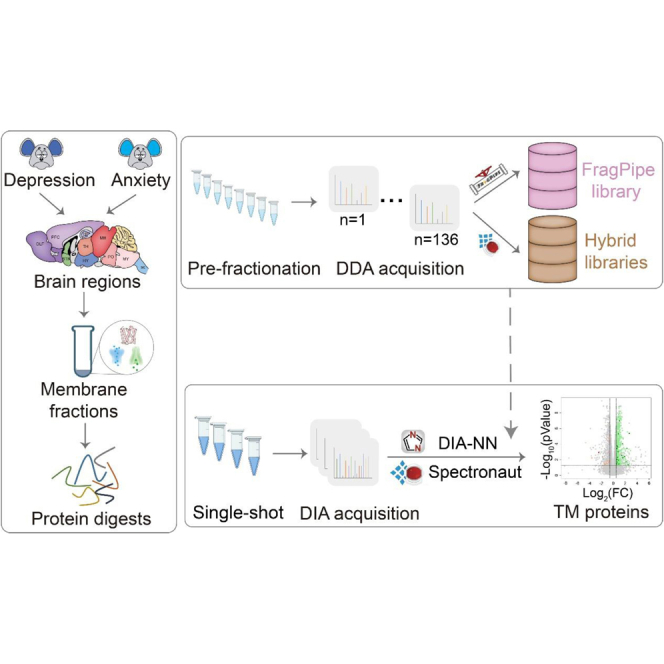

## In Brief

The establishment of a high-quality mouse brain transmembrane protein spectral library enables in-depth profiling of membrane receptors and other membrane proteins across various brain regions. Data-independent acquisition data analysis, combining multiple computational workflows all based on the spectral library, discovered a new regulator from the G protein–coupled receptor family which mediates depressive-like behaviors in an animal model. This library represents a valuable resource for brain transmembrane proteome mapping to facilitate the discovery of putative drug targets for treatment of psychiatric disorders.

Transmembrane (TM) proteins exert a wide range of physiological functions in the central nervous system (CNS) through mediating signal transduction, synaptic transmission, and maintaining homeostasis. Moreover, they play pivotal roles in the mechanisms underlying mental disorders or neurodegenerative conditions (1, 2, 3). TM proteins make up roughly 30% of mammalian proteome and approximately 60% of Food and Drug Administration-approved drug targets (4, 5). As the largest membrane protein family, G protein–coupled receptors (GPCRs) allow the nervous system to respond to external stimuli such as neurotransmitters and internal states (6). A large number of neuronal GPCRs are regarded as promising druggable targets for psychiatric disorders and neurodegenerative diseases (7, 8, 9, 10).

Multiple mouse brain regions have been systematically characterized at the molecular level through the application of diverse transcriptomics and imaging approaches. Resources such as the Allen Brain Atlas (11, 12, 13), the Human Protein Atlas (14, 15), and single-cell transcriptome atlases (16, 17, 18, 19) provide valuable insights into gene expression profiles, transcript/protein distribution, and the spatial organization of various cell types in the mouse brain.

In parallel to the near-complete profiling of gene expression in brain regions by transcriptomics, large-scale mass spectrometry (MS)-based proteomic surveys have been launched to map protein expression patterns across multiple regions of mouse and human brains (20, 21). The conventional MS method for proteomic data acquisition, data-dependent acquisition (DDA), is biased toward high-abundance soluble proteins in the cytosol and nucleus, resulting in a limited coverage of TM proteins. For example, in a global proteomic analysis, 1976 TM proteins and 61 GPCRs from more than 10,000 total proteins were detected from ten mouse brain regions (20). A similarly low coverage of TM proteins identification was reported in a global proteomic analysis of seven human brain regions, which detected 1926 TM proteins and 66 GPCRs from appropriately 9000 total proteins (21). Our recent study used a TM protein-optimized proteomic strategy which combined cell membrane fractionation with data-independent acquisition MS (DIA-MS) for accurate and consistent proteome quantification (22). In the previous work, we built a project-specific DDA library based on the data from multiregional brain tissues of resting-state mice. DDA data search with a conventional search engine (Proteome Discoverer) resulted in identification of 2459 TM proteins including 125 GPCRs in the original library.

To establish a deep mouse brain spectral library for comprehensive TM proteome profiling, herein, we combined DDA data previously acquired from the resting-state and depression-state mouse brains and newly acquired from brain regions of anxiety-state mice which comprised a total of 136 DDA runs. FragPipe was used to search the DDA data to build a spectral library that consists of 3043 TM proteins including 171 GPCRs. The significantly enlarged library contains 753 new TM proteins and 52 new GPCRs absent in the previous library. Equipped with this comprehensive TM protein-enriched spectral library, we analyzed DIA data collected from selected brain regions of two different chronic stress-induced mental disease models using three separate computational workflows incorporating Spectronaut or DIA-NN, which led to the identification of an expanded repertoire of dysregulated GPCRs in specific brain regions of disease models. Among them, we discovered an unreported GPCR regulator of depressive-like behaviors by *in vivo* pharmacological intervention. In addition, we compared the library-based DIA analysis results from the two models preferentially mimicking depression-like *versus* anxiety-like symptoms so as to reveal the landscape of TM proteome remodeling in the brain shared by or distinctive to these two psychiatric disorders.

## Experimental Procedures

### Experimental Design and Statistical Rationale

In this study, we aimed to provide a high-quality mouse brain TM spectral library to analyze the membrane receptor expression across multiple brain regions. To this end, each mouse brain region was collected from three mice per replicate for two disease models. In the anxiety model experiment, quadruplicate was prepared for each brain region (three regions in total) at the control or disease condition, and totally 24 mice were used for DDA and DIA MS data acquisition. In the depression model experiment carried out previously (22), triplicate was prepared for each brain region (11 regions in total) at the control or disease condition, and totally 30 mice were used for MS data acquisition. Individual replicates were analyzed with single-shot DIA-MS and peptide fractions from each brain region were analyzed with DDA-MS for spectral library generation.

For selection of differentially expressed (DE) proteins, a protein with a fold change >1.5 and adjusted *p*-value <0.05 given by Limma (version 3.54.2, with R version 4.2.1) was considered significantly changed. Any DE proteins with opposite trends of changes revealed by two different analysis workflows are removed from the final list.

### Mouse Brain Region Preparation

Subsequently, 8 to 16-week-old male C57BL/6 mice (Shanghai JieSiJie Laboratory Animal Co, Ltd) were housed under a 12-h light-dark cycle with ad libitum free access to water and food. The room temperature was 22 °C to 25 °C and the humidity was 40% to 50%. All procedures were performed according to the Institutional Animal Care and Use Committee at ShanghaiTech University and the National Institutes of Health guidelines (IACUC: 20231026001). The mice were euthanized with 2% chloral hydrate and rapidly dissected to get three brain regions (prefrontal cortex (PFC), hippocampus (HIP), and hypothalamus (HY)) for anxiety model mice (group and model groups). All the dissected regions were put into individual tubes, frozen on liquid nitrogen immediately, and stored at −80 °C before further analysis.

### Cell Membrane Proteins Digestion and Prefractionation

Cell membrane isolation, protein digestion, and prefractionation were according to our previous study (22). Each region was homogenized in the isolation buffer (30 mM Tris–HCl (pH 7.4), 0.1 mM EDTA, 0.5% bovine serum albumin, 300 mM sucrose, and 1× EDTA-free protease inhibitor (Roche)). The homogenate was centrifuged at 2500*g* for 10 min at 4 °C to remove the pellet. The supernatant was then centrifuged at 10,000*g* for 20 min at 4 °C. Subsequently, supernatant was subjected to ultracentrifugation at 160,000*g* for 1 h at 4 °C. The crude membrane was collected and washed using 1 M KCl, 100 mM Na_2_CO_3_, 100 mM Tris–HCl (pH 7.4) and ultracentrifuged for 1 h at 160,000*g* at 4 °C. The cell membrane was resolved in 5% sodium deoxycholate, 50 mM NH_4_HCO_3_ and heated 5 min at 95 °C. Protein concentration was determined by bicinchoninic acid assay (TIANGEN).

About 20 μg membrane protein of each replicate was reduced with 15 mM DTT, shaking at a thermomixer for 30 min at 56 °C, followed by 40 mM iodoacetamide to alkylated 30 min at room temperature. An additional 25 mM DTT was added to consume the excess iodoacetamide. Protein samples were diluted with 50 mM NH_4_HCO_3_ to a final 1% sodium deoxycholate concentration. Protein to enzyme = 50:1 (w/w) trypsin (Promega) was added and incubated 3 h at 37 °C, then protein to enzyme = 100:1 (w/w) trypsin was added and incubated overnight at 37 °C.

The protein digests from quadruplicate of each brain region were pooled and then fractionated using the high-pH RP spin column (Thermo Fisher Scientific) according to the manufacturer’s instructions. The peptides bound to the column were eluted into eight fractions with the acetonitrile (5–50%) step in a high-pH solution. The fractions were dried by vacuum centrifuge and store at −80 °C until liquid chromatography with tandem mass spectrometry (LC-MS/MS) analysis.

### DDA LC-MS/MS

The fractions were resolved in 0.1% formic acid and loaded on an in-house packed analytical column (75 μm × 20 cm x 1.9 μm) on an EASY-1200 coupled to Q-Exactive HF mass spectrometry (Thermo Fisher Scientific). Peptides were eluted in a 130 min linear gradient (5–35% solvent B, 0.1% formic acid in 80% acetonitrile) at 300 nl/min. The DDA acquisition settings were as follows: MS1 survey scan was 300 to 1650 m/z with resolution at 60,000 with automatic gain control (AGC) target 3 × 10^6^ with maximum injection time 20 ms. Top 15 precursors were selected for MS2 data acquisition. MS2 resolution was 15,000 with an AGC target set to 1 × 10^5^ with a maximum injection time of 25 ms and 1.4 m/z isolation window, and 27% NCE.

### DIA LC-MS/MS

Protein digests of brain regions were analyzed using Q-Exactive HF mass spectrometer with the DIA mode. The LC gradient setting was same as DDA. Precursors were fragmented in 22 variable windows covering a mass range of 300 to 1300 m/z (supplemental Table S5). The resolution was set at 120,000 for MS1 and 30,000 for MS2. AGC target was set to 3 × 10^6^ for both MS1 and MS2. Maximum injection time was set to 60 ms for MS1 and auto for MS2, and NCE was 25%, 27%, and 30%.

### Spectral Library Generation

We combined all 136 DDA runs previously acquired from the brain regions of resting-state mice, the depression model, as well as the newly acquired ones from the brain regions of the anxiety model to generate a spectral library using FragPipe (version 18.0) (23) software. Data search were conducted with a mouse Uniprot sequence database (Jun-2021, 55,366 Entries) supplemented with the indexed retention time (iRT) peptide sequences. The reversed protein sequences as decoys were initially added to the FASTA. The DDA files were searched using MSFragger (version 3.5) (24, 25) with the following parameters: trypsin as specific enzyme; C + 57.021464 as a fixed modification; M + 15.9949 and protein N-term +42.0106 as variable modifications; two missed cleavages allowed; peptide length 7 to 50; maximum fragment charge of two and minimum matched fragments of 4; 20 ppm for both precursor and fragment ions; MSBooster was applied for both spectra and retention time (RT) validation. Subsequently, verification of peptide-spectrum match and protein inference was performed using Percolator (26) and ProteinProphet (27), integrated within the software tool Philosopher (version 4.2.2) (28). The spectral library was generated using EasyPQP (version 0.1.29), with RT calibration based on iRTs and a Lowess fraction set to 0. The fragment types b and y ions were contained with 20 ppm mass tolerance. Furthermore, 1% false discovery rate (FDR) was filtered at protein and peptide levels. Meanwhile, we also generated two hybrid libraries by importing all the 136 DDA runs and 18 DIA runs from the depression model or 24 DIA runs from the anxiety model into Spectronaut (version 17.3) (29) against the same database with default settings. Trypsin was set as specific enzyme, carbamidomethyl (C) was set to fixed modification, and oxidation (M) and acetyl (protein N terminus) were variable modifications. Maximal two missed cleavages were allowed, and FDRs on peptide-spectrum match/peptide/protein levels were all set to 1%. Mass tolerance was set to default.

### DIA Data Analysis

#### Library-Based

The DIA files of three regions from depression or anxiety model were analyzed separately using DIA-NN (version 1.8.1) (30) with the FragPipe library or using Spectronaut (version 17.3) (29) with the FragPipe library or the hybrid library. The DIA-NN search parameters were set as below: Trypsin with one missed cleavage; peptide length 7 to 30, precursor charge state 1 to 4 and precursor mass range 300 to 1800 m/z; modifications, MS1 accuracy and scan window as default. Neural network classifier was double-pass mode, quantification strategy was Robust LC (high precision), cross-run normalization was RT-dependent. Precursor FDR was 1%.

The data search in Spectronaut were performed with default settings: Q value of precursor and protein cutoff set to 0.01; decoy generation method “Mutated”; quantification based on MS2 area; normalization strategy set to “Automatic”; Mass tolerance was set to default. The reports of protein quantification were exported and further analyzed.

#### Library-Free

The DIA files of three regions from depression model were analyzed using DIA-NN (version 1.8.1) (30) and Spectronaut (version 17.3) (29) with library-free mode. In the DIA-NN, the main search settings were the same with library-based mode except for spectral library imported. Protein FASTA was the mouse Uniprot sequence database (Jun-2021, 55,366 Entries) supplemented with the iRT peptide sequences. For directDIA analysis of Spectronaut, the settings of Pulsar search were default and same with hybrid library generation described above, and the parameters of DIA analysis were also default and same with library-based search.

### Comparison of Proteomic and Transcriptomic Datasets

The RNA-seq data were downloaded from the Human Protein Atlas. The ensemble IDs of RNA-seq were converted to gene names, and the matching rate was calculated based on all the genes present in both proteome and RNA-seq datasets.

### Pathway Enrichment

The pathway enrichment of DE TM proteins quantified in depression or anxiety model was performed with DAVID Bioinformatics Resources 6.8 (https://david.ncifcrf.gov/). The significant enrichment was set to adjusted *p*-value <0.01.

### Cell Type Enrichment

The cell type enrichment of DE TM proteins in brain regions of the depression or anxiety model was carried out with Enrichr (https://maayanlab.cloud/Enrichr/) (31). The significant enrichment was set to adjusted *p*-value <0.05.

### Mouse Model Procedures

In our previous study (22), we provided detailed procedures for establishing a depression model induced by chronic unpredictable mild stress. In the present study, to establish the anxiety model, we followed a published procedure by which mice were subjected to restraint stress in a plastic air-accessible cylinder for 2 h per day during the light phase and continuously treated for 10 days (32). Control mice were group-housed under comparable environmental conditions.

Subsequently, the mice underwent open field test (OFT) and elevated plus maze (EPM) tests. In the context of the anxiety model procedure, body weight of the mice was recorded.

### Compound Infusion

The mice were anesthetized with a mixture of isoflurane (1.0–1.5%) and oxygen (0.6–0.8 l per min). Then, they were securely immobilized on a stereotaxic device (RWD) with their skull surface exposed. All relevant equipment was sterilized with alcohol to ensure aseptic conditions. To implant the cannula, a 26-gauge double stainless-steel cannula (RWD) with 3.0 mm in length was gently inserted into the medial prefrontal cortex (mPFC). The coordinates for this implantation were as follows: anterior-posterior, +1.60 mm from the bregma; medial-lateral, ±0.35 mm lateral to the sagittal suture; and dorsal-ventral, −1.70 mm from the dura mater.

Prosaptide TX14(A) was synthesized from Changzhou Kanglong Biotech Ltd. Following the surgical procedure, all mice were allowed to recover for at least 1 week. A 26-gauge double internal injector with a 3-mm projection was inserted into the guide cannula before the compound infusion. Substances, including prosaptide TX14(A) at doses of 1.25 μg or 5 μg per side (500 nl), ketamine at 5 μg per side (500 nl), 3% dimethyl sulfoxide (500 nl), or 0.9% saline (500 nl) were delivered at a controlled rate of 200 nl/min through the injector. After a 5-min interval, the injector cannula could be safely removed.

### Animal Behavioral Tests

All mice were 10 to 16-week-old and the behavioral tests were performed in the specific time 14:00 to 21:00 PM. For certain tests, sucrose preference test (SPT), OFT, and EPM had to be carried out during dark phase, however, forced swim test (FST) and tail suspension test (TST) were conducted during the light phase. Before FST, the mice were allowed to swim for 10 min each day for a period of 2 days.

#### Sucrose Preference Test

To facilitate adaptation, mice were individually housed and provided with access to a bottle containing 2% sucrose solution, in conjunction with a bottle of water, for a duration of 3 days. Subsequently, they underwent 24 h of water deprivation. During the test, the mice were presented with two identical bottles, one was 1% sucrose solution and the other was water. After 2 h, the consumption of sucrose solution or water was measured through the weight of two bottles. To prevent habituation, the positions of the two bottles were alternated every 24 h during the adaptation period and every 1 h during the test period. Sucrose preference was calculated as the ratio of the weight of sucrose consumption to the summed weight of sucrose and water consumption during the 2-h test.

#### Open Field Test

Mice were placed in the center of a white open-field chamber (30 cm × 30 cm × 40 cm) and their movement was monitored by a video camera (Shanghai Jiliang Software Technology Co Ltd) above the chamber for 10 min in the arena after anxiety model establishment, whereas behavior was monitored for 5 min after substance infusion into the mPFC of naïve or depression model mice. The duration of time cost in the central area of the chamber serving as a gauge of anxiety or depression behavior. The total distance traveled was automatically calculated.

#### Tail Suspension Test

Four mice were secluded from both auditory and visual stimuli and suspended in a digital recording device. To prevent the mice from contacting or climbing on any object, they were hung up at a height of 50 cm from the floor using a tail strap that was fixed 1 cm from the tip of the tail. The activities of the mice were recorded for 6 min. The duration of immobility, specifically recorded during the last 4 min of the period, was subjected to analysis.

#### Forced Swim Test

Mice were placed in a transparent glass cylinder measuring 40 cm in height and 20 cm in diameter. The cylinder was filled with water at temperature of 26 °C to 27 °C, and the depth was adjusted to ensure that the mice were unable to reach the bottom with their tails or hind limbs. During the test, mice were given a 6-min period to swim. The immobility time was measured by the floating of mice in the water without making significant struggling movements, and by performing only the minimal movements necessary to keep their heads above the water. The final 4 min of the test period was further calculated as the total immobility time.

#### Elevated Plus Maze

The maze device, a height of 74 cm above the floor, included two opposing open arms and two enclosed arms, and both are 35 cm × 6 cm. The enclosed arms were extended from a central platform (6 cm × 6 cm). In the test, mice were put into the central area of the maze and faced an open arm. The behaviors were recorded for 10-min using a video-tracking device (Shanghai Jiliang Software Technology Co). The entries into the open arms and the time spent in the open arms were monitored and analyzed.

### Statistical Tests

The behavioral data analysis was performed by GraphPad Prism (GraphPad Software, Inc; https://www.graphpad.com) with paired or unpaired *t* test and one-way or two-way analysis of variance (followed by a Bonferroni’s multiple comparison test). Data were showed as mean ± SEM. Significance was defined as ∗*p* <0.05, ∗∗*p* <0.01, ∗∗∗*p* <0.001, and ∗∗∗∗*p* <0.0001.

## Results

### Generation of the Mouse Brain TM Protein Spectral Library

**Fig. 1:**
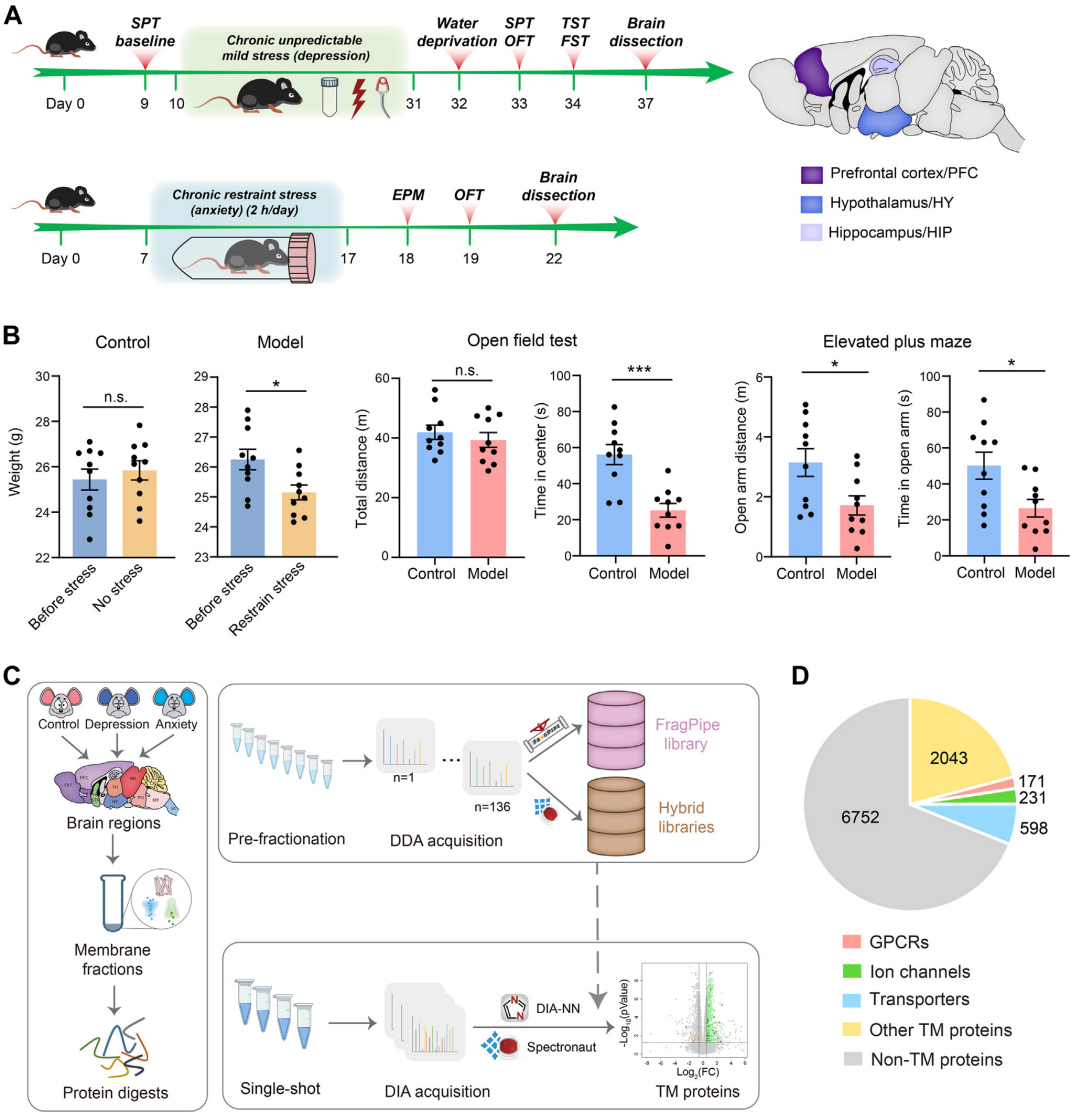
**Overview of the spectral library generation and DIA data mining.***A*, overall workflow for establishing depression mouse model induced by chronic unpredictable mild stress and anxiety mouse model induced by chronic restraint stress, with focused analysis on three brain regions associated with mental disorders. *B*, behavioral tests for an anxiety mouse model showed weight loss, shorter time in center in OFT, and shorter distance and time in open arm in EPM. *C*, workflow of spectral library generation, involving membrane isolation, protein digestion, prefractionation, and analysis of resting-state and disease-state mouse brain regions. A total of 136 DDA files were used to build spectral libraries with FragPipe or Spectronaut. DIA data were mined using three analysis workflows with different libraries. *D*, number of protein identifications (IDs) in the library built with FragPipe. DIA, data-independent acquisition; EPM, elevated plus maze; OFT, open field test.

In our previous study, we constructed a project-specific library by prefractionating membrane protein extracts from ten brain regions of resting-state mice. In addition, we established a TM protein-optimized proteomic pipeline to profile the TM proteome regulation in the brain of a depression mouse model induced by chronic unpredictable mild stress (22). In this study, to expand the spectral library tailored to proteomic studies of mental diseases, we established an anxiety mouse model induced by chronic restraint stress (32) to further increase the TM proteome coverage that is achievable by DIA-MS analysis. Mice were subjected to 2-h restraint stress per day and continuously treated for 10 days which would induce anxiety-like symptoms (32) (Fig. 1*A*). As expected, weight loss was observed in the anxiety model procedure, and results from two standard behavioral tests (OFT and EPM tests) were indicative of an anxiety-like phenotype (32) (Fig. 1*B*). For the anxiety model and control mice, we dissected three brain regions (PFC, HIP, and HY) with indispensable roles in mood regulation and psychiatric disorder development (33, 34, 35, 36) (Fig. 1*A*). Subsequently, we carried out cell membrane isolation, protein extraction, and digestion for LC-MS/MS analysis as detailed in Experimental Procedures (Fig. 1*C*). For each brain region, we prefractionated the pooled replicates and analyzed them by DDA-MS. Meanwhile, all protein digests from individual brain regions were subjected to single-shot DIA-MS analysis (Fig. 1*C*).

We then combined all DDA data of 136 runs previously acquired from resting-state mice and the depression mouse model and newly acquired from the anxiety model to construct a spectral library by data search with FragPipe. This FragPipe library comprised a total of 3043 TM proteins including 171 GPCRs, 231 ion channels and 598 transporters (Fig. 1*D*). Additionally, we built two hybrid libraries using Spectronaut based on the same DDA dataset and an additional collection of 18 DIA runs from the depression model or 24 DIA runs from the anxiety model.

Comparing the comprehensive FragPipe library with our previous project-specific DDA library, we observed increased proteome coverages (22.5% increase of total proteins, 23.7% increase of TM proteins, and 36.8% increase of GPCRs) (supplemental Fig. S1*A*). Moreover, the overlap between the FragPipe and hybrid libraries ranged from 56.5% to 62.4% for total proteins and TM proteins (supplemental Fig. S1*B*). Therefore, the high proportions of unique proteins (2.4%–18.6%) present in different libraries prompted us to investigate their influences on TM proteome profiling with DIA-MS analysis.

### Brain Region Proteome Profiling for the Depression Model with Different Workflows

As our previous study revealed considerable impact of different software packages and informatics workflows on the DIA data analysis results (37), we sought to evaluate two widely used software tools, DIA-NN and Spectronaut, in utilizing the FragPipe library or the hybrid library for protein identification and quantification with DIA data from three brain regions (PFC, HIP, HY) of the depression model.

**Fig. 2:**
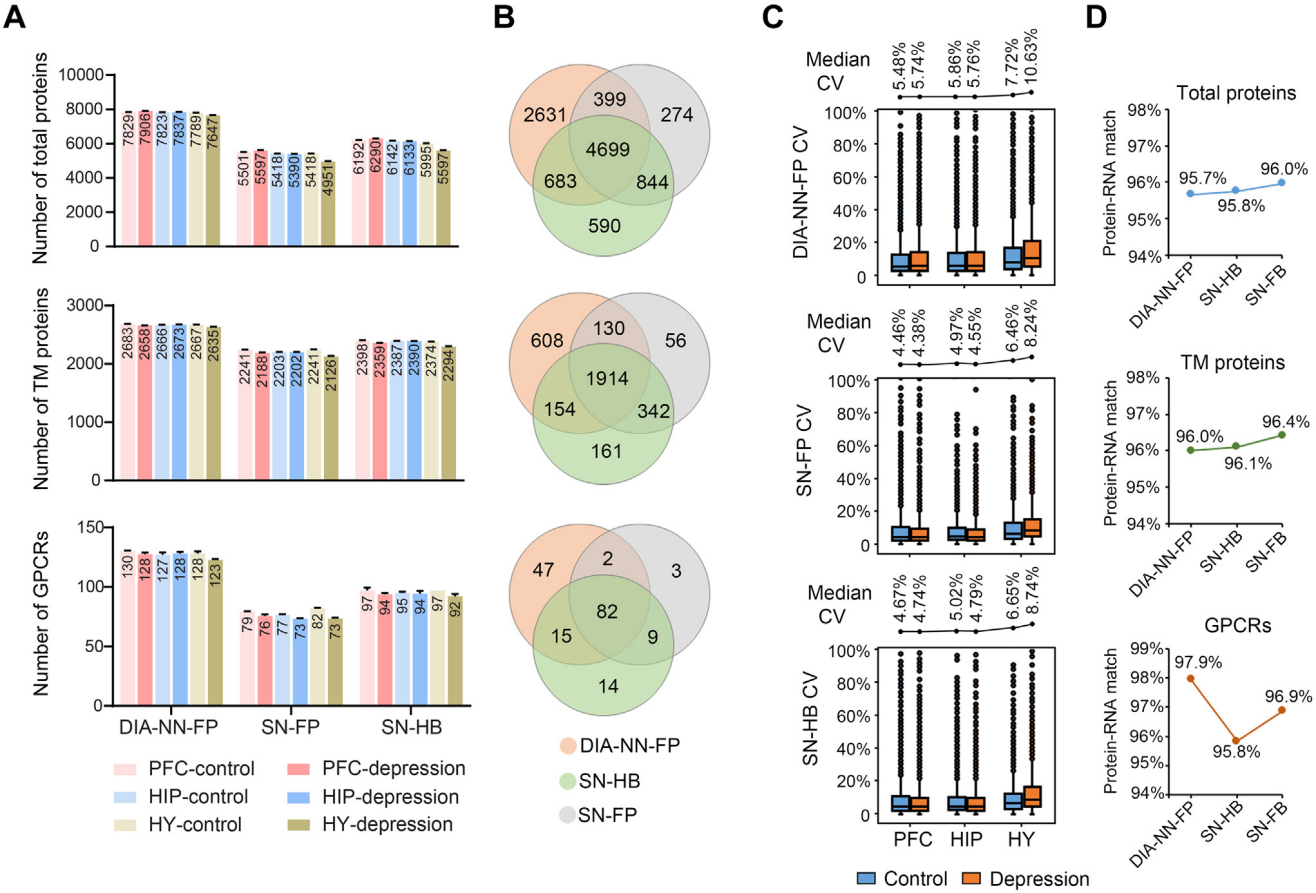
**Proteome identification and quantification performance in the depression model data analysis with three workflows.***A*, average number of protein IDs in each brain region with three analysis workflows (DIA-NN-FP, DIA-NN with FragPipe library; SN-FP, Spectronaut with FragPipe library; SN-HB, Spectronaut with hybrid library). *B*, Venn plots of the total proteins, TM proteins or GPCRs reported by three workflows. *C*, CV distribution of TM proteins from specific brain regions, indicating the high reproducibility of three analysis workflows. *D*, protein-to-mRNA matching rates for total proteins, TM proteins, and GPCRs reported by three workflows. DIA, data-independent acquisition; GPCR, G protein–coupled receptor; TM, transmembrane.

Specifically, we processed the brain region DIA data using three different library-based workflows: DIA-NN with the FragPipe library (DIA-NN-FP), Spectronaut with the FragPipe library (SN-FP), and Spectronaut with the hybrid library (SN-HB). DIA-NN-FP yielded the highest proteome coverage by reporting an average of 7805 protein groups containing 2664 TM proteins and 127 GPCRs (Fig. 2*A*, and supplemental Table S1). Spectronaut with the FragPipe library reported 5379 protein groups including 2200 TM proteins and 76 GPCRs, a coverage lower than the same tool with the hybrid library which reported 6058 protein groups including 2367 TM proteins and 95 GPCRs (Fig. 2*A*, and supplemental Table S1). In comparing the three data analysis workflows, we observed approximately 47.7% to 56.9% overlap in the TM and GPCR protein identifications (Fig. 2*B*). Of note, each workflow also gave rise to its own unique set of protein groups, with DIA-NN-FP generating the highest proportions of exclusively identified TM and GPCR proteins (Fig. 2*B*). The median of CV for TM proteins over three independent replicates of each brain region as determined by three workflows were all below 10% except for one condition with DIA-NN-FP, indicating overall excellent quantification consistency (Fig. 2*C*). Moreover, the reproducibility achieved with Spectronaut (median CVs of 4.38%–8.24% with the FragPipe library; median CVs of 4.67%–8.74% with the hybrid library) was slightly better than the results of DIA-NN-FP (median CVs of 5.48%–10.63% with the FragPipe library) for all the brain regions (Fig. 2*C*). Importantly, our DIA TM proteome profiling with three workflows was corroborated by transcriptome mapping given that more than 95% of identified proteins were also detected at the transcript level in the mouse brain (95.7%–96.0% matching rates for total proteins; 96.0%–96.4% for TM proteins; 95.8%–97.9% for GPCRs) (Fig. 2*D*).

Considering that library-free approaches are gaining more popularity in DIA data analysis due to their faster speed and flexibility (37, 38), we then processed the same DIA data using two additional workflows: DIA-NN in the library-free mode and Spectronaut in the directDIA mode. Protein identification and quantification results with these workflows are summarized in supplemental Fig. S2. Of note, both library-free workflows yielded lower numbers of total proteins, TM proteins, and GPCRs than the respective library-based workflows (supplemental Fig. S2, *A* and *B*). Spectronaut gave rise to slightly higher quantification reproducibility than DIA-NN across the three brain regions for the library-free workflows (supplemental Fig. S2*C*).

Taken together, in analyzing DIA data from the brain regions of the depression model, DIA-NN with a high-quality and comprehensive spectral library yielded the deepest proteome coverages with the highest numbers of unique identifications among all tested library-based and library-free workflows, whereas Spectronaut achieved slightly better reproducibility of quantification. In addition, considering the typically large variations of biological replicates collected for each brain region, data completeness is acceptable and consistent with previous reports (37, 39, 40) for both library-based and library-free workflows with protein missing values in the range of 5.9%-11.1% (supplemental Fig. S2*D*).

### DE Protein Analysis for the Depression Model with Different Workflows

**Fig. 3:**
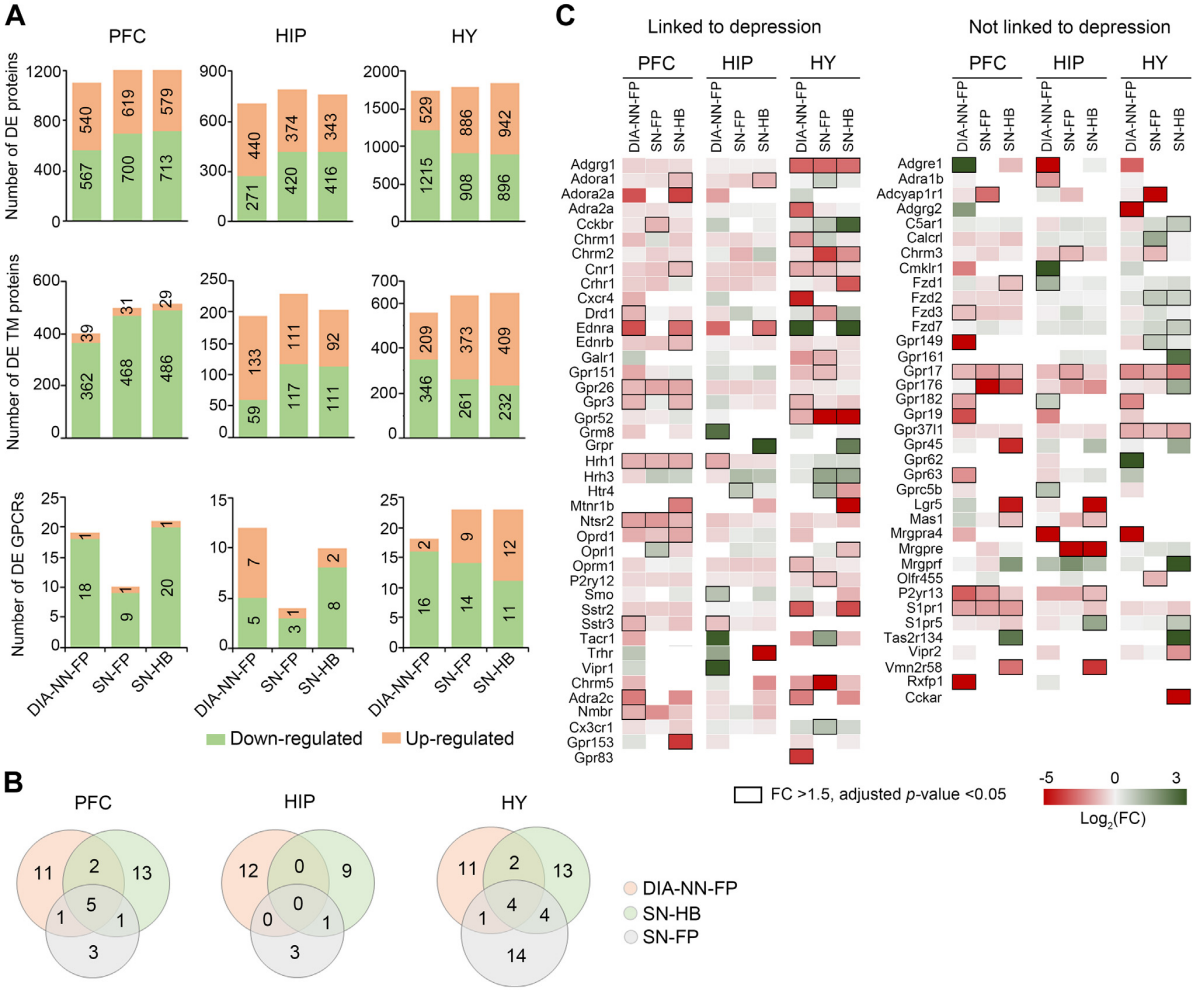
**DE protein analysis in three brain regions of the depression model.***A*, number of DE proteins, TM proteins, and GPCRs (FC >1.5, adjusted *p*-value <0.05) in three regions reported by three analysis workflows. *B*, overlap of DE GPCRs discovered by three workflows. *C*, heatmap of 78 DE GPCRs in specific brain regions reported by three analysis workflows. DE GPCRs linked (*left*) or never linked (*right*) to depression in literature are annotated. DE proteins with significant changes are indicated by *black boxes*. DE, differentially expressed; FC, fold change; GPCR, G protein–coupled receptor; TM, transmembrane.

To assess the utility of our library-based DIA proteomics in discovering potential regulators of psychiatric disorders, we compared DE proteins in each brain region of the depression model which were selected based on search results from three library-based workflows. Interestingly, similar coverages of DE TM proteins were obtained by three analysis workflows (Fig. 3*A*, and supplemental Table S3), yet only with moderate overlapping (11.1%–25.5% of shared protein identifications by three workflows) (supplemental Fig. S3*A*). Notably, the majority of DE TM proteins in PFC (90.3%–94.4%) were downregulated in the disease model while their upregulation and downregulation were more balanced in HIP and HY regions (Fig. 3*A*). Given our particular interest in mining the GPCR family in search of molecular regulators, we further examined DE GPCRs in each brain region of the depression model. DIA-NN-FP reported a set of DE GPCRs vastly different from that given by Spectronaut (Fig. 3*B*), with only 8.2% to 13.9% overlapping in PFC and HY regions and no overlapping in the HIP region. In addition, two Spectronaut-based workflows yielded 7.7% to 24.0% overlapping of DE GPCRs and the SN-HB workflow yielded more regulated DE proteins than SN-FP. Within a total of 78 DE GPCRs from different brain regions using three data analysis workflows, 39 regulated receptors are reported by the DIA-NN-FP workflow, 31 by SN-FP and 42 by SN-HB workflows.

Among the DE GPCRs identified in this study, 41 have been reported to be regulators of depressive-like behaviors in animal models and thus serve as potential antidepression targets whereas 37 are never linked to depression in functional studies (Fig. 3*C*, and supplemental Table S4). For example, G protein–coupled receptor 26 (Gpr26) is expressed in many regions of mouse brain (olfactory area, amygdala, HIP, and cortex) (41, 42). All three workflows reported Gpr26 to be significantly downregulated in PFC (>1.6-fold, adjusted *p* < 0.05) and moderately downregulated in HIP (>1.3-fold, adjusted *p* < 0.05) of depression mice, in line with the finding that *Gpr26* KO mice displayed increased levels of anxiety- and depression-like behaviors (41).

Surprisingly, 26 disclosed GPCR regulators are discovered by one specific workflow. For example, somatostatin receptor type 3 (Sstr3) was downregulated in the PFC and HIP brain regions and muscarinic acetylcholine receptor M1 (Chrm1) was found to be downregulated in HY region, as revealed only by the DIA-NN-FP workflow. Intriguingly, infusion of somatostatin receptor type 3 agonist resulted in the antidepressant-like effect (43), whereas genetic deficiency (44) or antagonism (45) of M1 receptor caused an antidepressant- or anxiolytic-like behavior. On the other hand, the SN-HB workflow exclusively revealed downregulation of both melatonin receptor 1B (Mtnr1b) and delta-type opioid receptor (Oprd1) in PFC region. Consistently, mice with gene deficiency of Mtnr1b (46) or Oprd1 (47) displayed anxiogenic- and depressive-like responses, and agonist of Oprd1 produced antidepressant-like effects in animal models (48). Additionally, we found that seven DE GPCRs were only contributed by the SN-FP workflow. For example, Galanin receptor type 1 (Galr1) and muscarinic acetylcholine receptor M5 (Chrm5) were downregulated in HY region. Interestingly, knocking down *Galr1* (49) or inhibiting M5 receptor (50) attenuated the depressive- and anxiogenic-like behavioral phenotypes, and stimulation of Galr1 led to depressive-like behaviors (51).

Taken together, our results suggested that the three data analysis workflows utilizing different libraries possessed distinct characteristics and complemented each other, particularly in the discovery of regulated GPCRs. Thus, integration of three analysis workflows resulted in a comprehensive catalog of DE TM and GPCR proteins in the analysis of depression model.

### Brain Region Proteome Profiling and DE Protein Analysis for the Anxiety Model

**Fig. 4:**
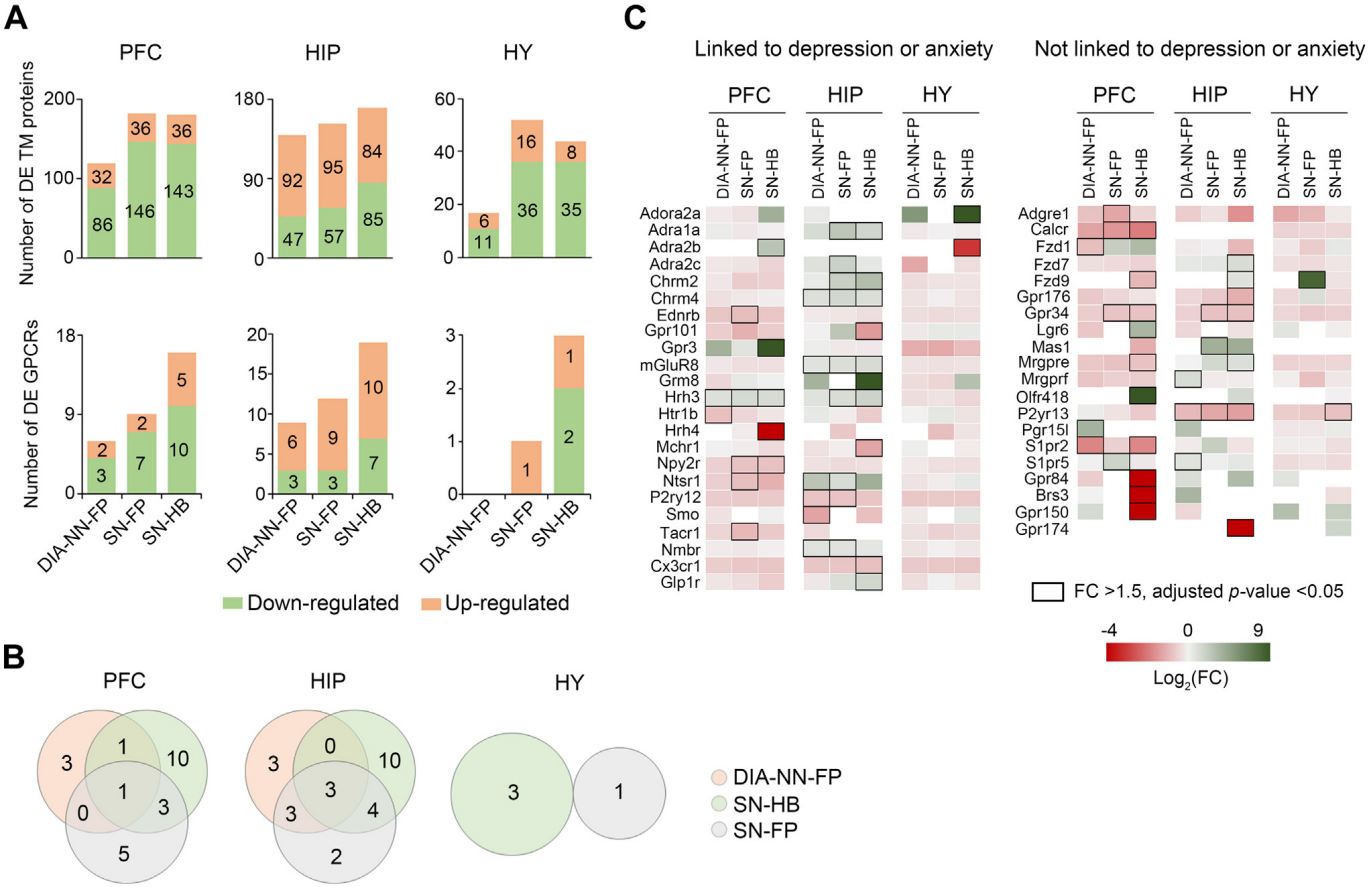
**DE protein analysis in three brain regions of the anxiety model.***A*, number of DE TM and GPCR proteins (FC >1.5, adjusted *p*-value <0.05) in three regions reported by three analysis workflows. *B*, overlap of DE GPCRs discovered by three workflows. *C*, heatmap of all 43 DE GPCRs in specific brain regions reported by three analysis workflows. DE GPCRs linked (*left*) or never linked (*right*) to depression or anxiety in literature are annotated. DE proteins with significant changes are indicated by *black boxes*. DE, differentially expressed; FC, fold change; GPCR, G protein–coupled receptor; TM, transmembrane.

Owing to our previous experience with the depression model, we chose to implement three complementary analysis workflows in the data analysis for the anxiety model. Overall, the DIA-NN and Spectronaut workflows gave rise to a comparable coverage of the TM proteome from three brain regions (supplemental Table S2). In terms of DE proteins, we noted that more DE TM or GPCR proteins were detected by SN-FP and SN-HB workflows in the anxiety model (Fig. 4*A*, and supplemental Table S3), yet they also showed compositions highly complementary to those detected by the DIA-NN-FP workflow (Fig. 4*B*, and supplemental Fig. S3*B*). As mentioned before, combining the outputs from both DIA-NN and Spectronaut workflows can enhance the identification of potential GPCR targets associated with mental diseases. Indeed, our analysis of the anxiety data by three workflows collectively revealed a total of 43 DE GPCRs in three brain regions of the anxiety model, with 14, 19, and 29 contributed by DIA-NN-FP, SN-FP, and SN-HB workflows, respectively.

Among these DE GPCRs in specific brain regions, 23 have known implications in depression or anxiety-related psychiatric disorders (supplemental Table S4). For example, both smoothened homolog (Smo) and 5-hydroxytryptamine receptor 1B (5-HT_1B_R) were downregulated in HIP and PFC regions, respectively, as revealed only by the DIA-NN-FP workflow. Consistently, *Smo* KO mice displayed increased anxiety- or depression-like behaviors (52), and an 5-HT_1B_R agonist produced an antidepressant effect in the FST in mice (53). On the other hand, nine DE GPCRs were only contributed by the SN-HB workflow. For instance, adenosine receptor A2a (A_2A_R) showed upregulation in HY region, whereas 5-hydroxytryptamine receptor 4 (5-HT_4_R) exhibited significant downregulation in PFC region, consistent with the findings that activation of A_2A_R was associated with increased depressive-like symptoms (54), and antagonists of A_2A_R (55) or agonists of 5-HT_4_R (56) showed antidepressant effects. Additionally, the SN-FP workflow exclusively revealed upregulation of Alpha-2C adrenergic receptor (α_2C_-AR) in HIP region, and downregulation of substance-P receptor (Tacr1) in PFC region. Intriguingly, genetic deficiency or antagonism of α_2C_-AR (57, 58) and Tacr1 (59, 60) displayed antidepressant and antipsychotic-like effects.

Furthermore, we discovered 20 DE GPCRs that have not been previously linked to these psychiatric disorders, warranting further investigation (Fig. 4*C*). Therefore, DIA data analysis of the two mental disease models underscores the advantage of merging complementary data analysis workflows to increase our chances of discovering GPCR regulators which may serve as potential drug targets associated with mental diseases.

### Comparison of TM Proteome Profiles for Two Psychiatric Disorder Models

**Fig. 5:**
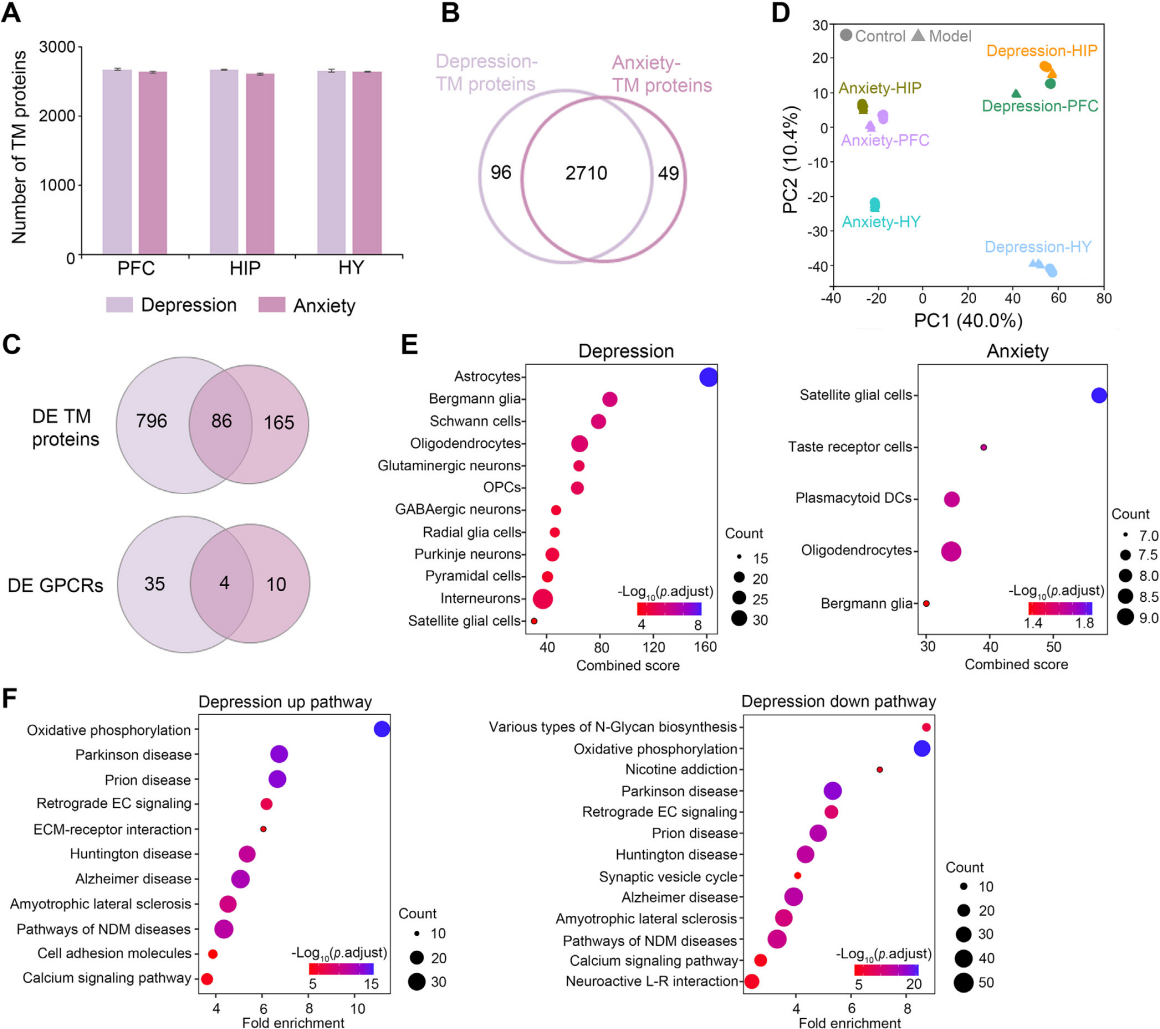
**Comparison of TM proteome regulation in depression model *versus* anxiety model.***A*, number of total TM proteins quantified in three regions of depression and anxiety models. *B*, Venn plot showing the overlap of TM proteins profiled in two mouse models. *C*, Venn plots of DE TM or GPCR proteins from three brain regions of two models. *D*, PCA plot of three regional expression of total TM proteins demonstrates the clustering of control, depression or anxiety groups, with distinct separation of two models. *E*, significantly enriched cell types (adjusted *p* <0.05) of DE TM proteins from two models. *F*, significantly enriched pathways (adjusted *p* <0.01) of upregulated or downregulated TM proteins from the depression model. No significant enriched pathways are obtained for anxiety model. OPCs, oligodendrocyte progenitor cells; plasmacytoid DCs, plasmacytoid dendritic cells; retrograde EC signaling, retrograde endocannabinoid signaling; pathways of NDM diseases, pathways of neurodegeneration−multiple diseases. DE, differentially expressed; GPCR, G protein –coupled receptor; PCA, principal component analysis; TM, transmembrane.

In this study, we analyzed the TM proteome profiles in the same set of brain regions (PFC, HIP, and HY) from two mental disease models which differ in the type, strength, and duration of stressors received by the animals (Fig. 1*A*). These two models are believed to engage distinct pathologic mechanisms and molecular landscape underlying depressive or anxiety disorders (61, 62, 63). To illustrate the similarity and difference in the proteome signatures between the two disease models, we compared the TM proteome profiles yielded by the DIA-NN-FP analysis workflow. An average of 2664 and 2624 TM proteins was quantified across three brain regions in the depression and anxiety models, respectively (Fig. 5*A*). We observed a high degree of overlap in the proteome composition, with 95% of TM proteins being shared by two models (Fig. 5*B*).

However, with respect to TM proteome regulation, we found considerably more DE TM proteins from the depression model than the anxiety model (882 DE TM proteins from depression *versus* 251 from anxiety) (Fig. 5*C*). Furthermore, the DE GPCRs identified in brain regions of the depression model were nearly completely distinct from those of the anxiety model. Specifically, 39 DE GPCRs were discovered in depression whereas 14 were found in anxiety (Fig. 5*C*), with only one DE GPCR from the HIP region in common (supplemental Fig. S4). Notably, among the four DE GPCRs identified in both models (Fig. 5*C*), Smo has established associations with depressive or anxiety disorders (52), and our previous study has revealed the role of neuromedin B (NMB) receptor (NmbR) in developing depressive-like symptoms in the animal model (22).

The distinctive molecular architectures in the brain regions between two models are also manifested in the principal component analysis plot based on all quantified TM proteins. All control and disease groups of the depression model are clearly segregated from those of the anxiety model. Within each model, we observed more significant separation between control and disease groups for the PFC region than for HY and HIP regions, indicating the largest perturbation of the TM proteome in PFC by stresses implemented in both models (Fig. 5*D*).

Cell type enrichment analysis of DE TM proteins from depression and anxiety model demonstrated the associations with most nonneuronal cells, including astrocytes, Bergmann glia, oligodendrocytes, satellite glial cells, and more (Fig. 5*E*), which play important roles in maintaining homeostasis in CNS and provide metabolic support to neurons (64). This result underscores the significance of these cells in the context of mental health and suggests a potential link between these nonneuronal cells and the observed TM proteome remodeling associated with psychiatric disorders.

The group of 350 upregulated TM proteins identified in depression model was enriched in 11 pathways, mainly involving oxidative phosphorylation, retrograde endocannabinoid signaling, calcium signaling pathways, and extracellular matrix-receptor interaction (Fig. 5*F*). On the other hand, the group of 660 downregulated TM proteins in depression model was enriched in 13 pathways, which, in addition to the above pathways, included various types of N-glycan biosynthesis, synaptic vesicle cycle, and neuroactive ligand-receptor interaction pathways (Fig. 5*F*). Notably, either upregulated or downregulated TM proteins were also enriched in pathways associated with the development of neurodegenerative diseases such as Parkinson disease, Huntington disease, and Alzheimer disease, which supported a proposed speculation of shared molecular mechanisms between neurodegeneration and depression (65) (Fig. 5*F*). In accordance with the much less proteome perturbation, we did not find any significant enriched pathways for anxiety model, suggesting its overall milder stress response than depression model. In comparing the TM proteome profiles obtained from the SN-HB analysis workflow (supplemental Fig. S5), we observed similar results to the DIA-NN-FP workflow, thereby strengthening the reliability of these findings.

### Discovery of a New Regulator of Depression from the GPCR Family

Among the 37 DE GPCRs, newly identified from the depression model in this study yet without any implication in depression, G protein–coupled receptor 37-like 1 (Gpr37l1) attracted our attention mainly due to its enriched expression in the CNS especially in astrocytes and certain oligodendrocyte precursors (66). Gpr37l1 was found to be significantly downregulated in HY (>1.5-fold, adjusted *p* < 0.01) and modestly downregulated in PFC and HIP of depression mice (>1.2-fold, adjusted *p* < 0.05) relative to control (supplemental Table S3).

**Fig. 6:**
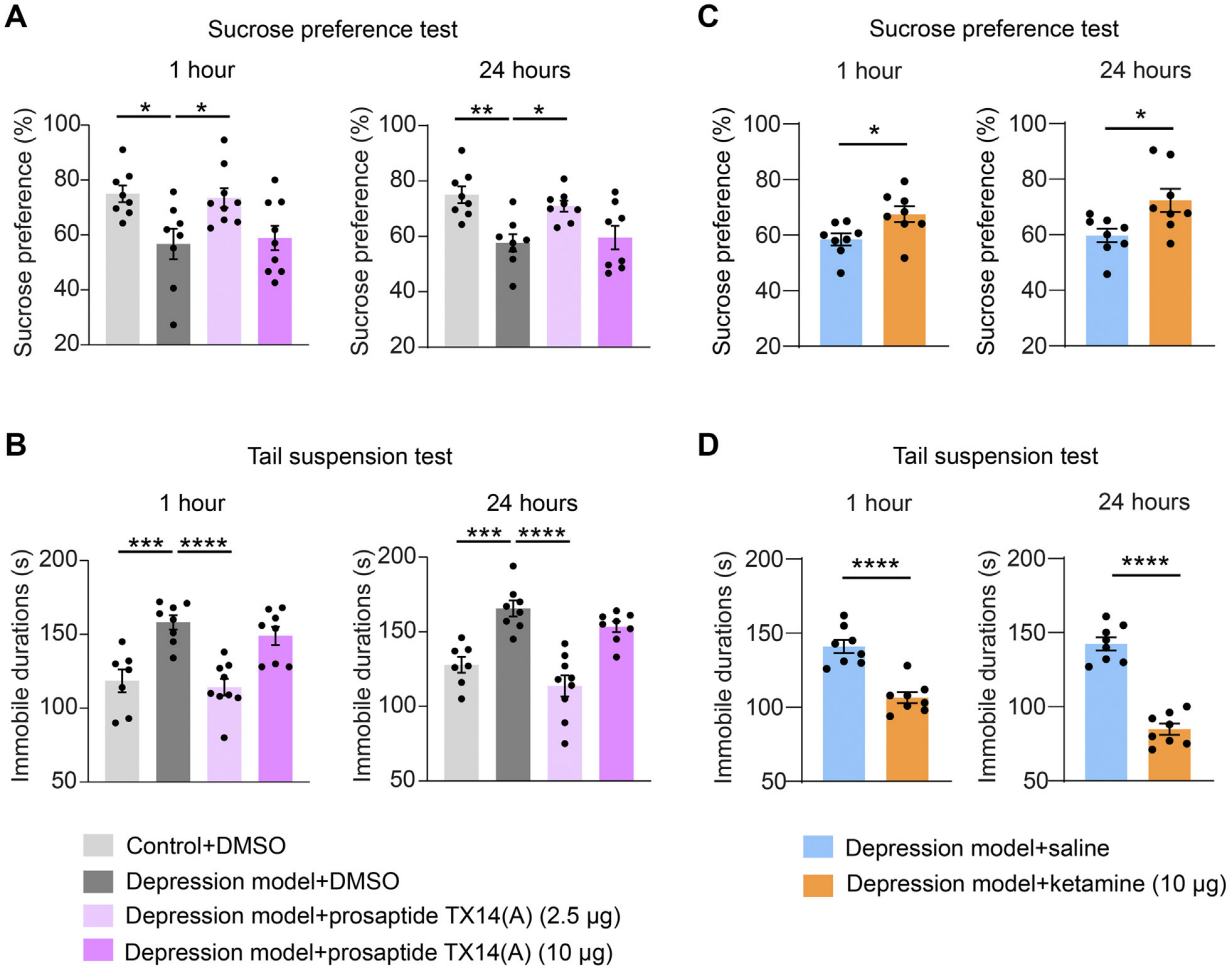
**Behavior tests of a GPCR agonist and ketamine in a mouse depression model.***A* and *B*, antidepressant effects of stereotactic infusion of prosaptide TX14(A) (1.25 and 5 μg each side) into mPFC at 1 and 24 h after infusion as measured in SPT (*A*) and TST (*B*). *C* and *D*, antidepressant effects of an antidepression drug ketamine (5 μg each side). Behaviors were measured in SPT (*C*) and TST (*D*) at 1 h and 24 h postinfusion. Data are mean ± SEM, ∗*p* <0.05, ∗∗*p* <0.01, ∗∗∗*p* <0.001, ∗∗∗∗*p* <0.0001. GPCR, G protein–coupled receptor; mPFC, medial prefrontal cortex; SPT, sucrose preference test; TST, tail suspension test.

To assess the impact of modulating Gpr37l1 activity on acute depressive-like behaviors in mice, we conducted stereotactic infusion of a Gpr37l1 agonist prosaptide TX14(A) into the mPFC of naïve mice. The result showed a reduction in immobility time in mice at 24 h after infusion of a low dose of prosaptide TX14(A) (1.25 μg each side) in TST, indicating an enduring antidespair effect (supplemental Fig. S6*A*). Infusion of prosaptide TX14(A) did not induce depressive behavior in the FST (supplemental Fig. S6*B*). Furthermore, the infusion of prosaptide TX14(A) (1.25 μg and 6.25 μg each side) into mPFC led to an increase in the total distance traveled in the OFT but no changes in the center zone time, suggesting an antianxiety effect (supplemental Fig. S6*C*). Next, to further verify the antidepressive effect of prosaptide TX14(A) in a chronic stress-induced depression model, we infused prosaptide TX14(A) into mPFC and measured multiple behavioral tests. We observed that depressive-like behaviors were attenuated in the SPT and TST at 1 and 24 h after infusion (Fig. 6, *A* and *B*).

As ketamine is a fast-acting antidepressant drug approved for the treatment of major depressive disorder (67, 68), we infused ketamine (5 μg each side) into the mPFC and measured the effects on SPT, TST, and FST at 1 h and 24 h postdrug infusion (Fig. 6, *C* and *D*, and supplemental Fig. S7*B*). Remarkably, in comparison to ketamine, prosaptide TX14(A) displayed a similar effect in reversing depressive-like behaviors at both 1 and 24 h (Fig. 6). Besides, no change was observed in the general locomotion or anxiety behavior in the OFT after prosaptide TX14(A) or ketamine treatment at 24 h postinfusion (supplemental Fig. S7, *A* and *C*). Additionally, there was no sustained antidepressant effects in TST and SPT after 3 days of prosaptide TX14(A) infusion (supplemental Fig. S7*D*). These results indicated that the prosaptide TX14(A) produced a rapid antidepressant response *in vivo* possibly by activating Gpr37l1, and its pharmacological effects are very similar to ketamine.

## Discussion

Here, we established a comprehensive brain TM protein spectral library including more than 3000 TM proteins and 171 GPCRs that are expressed across multiple mouse brain regions. While library-free approaches can provide the merits of flexibility and convenience for DIA analysis, a deep and high-quality library is still a cornerstone for robust and reproducible DIA data mining to achieve accurate and near-complete proteome profiling (37, 38).

TM proteins are characterized by their high hydrophobicity, low abundance, and relatively fast turn-over, which have posed a significant challenge for global proteomic analysis which favors cytosolic and nuclear protein mapping in both cells and tissues (69, 70). Our approach addresses this challenge through a technical solution by combining cell membrane isolation, DIA-MS, and the use of a TM protein-enriched spectral library to enhance the TM proteome coverage, especially for the GPCRs in brain tissues. Furthermore, by integrating three data analysis workflows and leveraging spectral libraries built with different tools, our study quantified a total of 172 GPCRs in the depression model and 176 GPCRs in the anxiety model from three brain regions known to mediate symptoms of psychiatric disorders. Although we expect to find distinct DE GPCRs using informatics workflows using two software tools (DIA-NN *versus* Spectronaut), it was surprising to see Spectronaut with two types of libraries (FragPipe library *versus* hybrid library) yielded a significant fraction of DE GPCRs unique to either library (Figs. 3 and 4). More importantly, in the data analysis of the depression model, 17.1% to 26.8% of disclosed regulators of depression identified by our study were exclusively reported by one specific workflow (Fig. 3*C*). Most of these workflow-specific DE GPCRs showed the same trends of regulation by at least two library-based workflows, yet they were only selected by one workflow under our defined DE protein criteria (Fig. 3*C*). This result indicated that small differences in protein quantification by different analysis workflows would affect the fold change and *p*-value determination and ultimately influence DE protein selection. Given that 48.1% of these workflow-specific DE GPCRs turn out to be reported functional regulators of depression, we chose to combine DE protein lists generated by multiple complementary workflows to expand the pool of candidate targets. However, such a combination strategy may also cause a potential increase of false positives, thus requiring orthogonal validation and functional evaluation.

GPCRs have been a focus of enduring interest as pharmacological targets for neuropsychiatric disorders or other diseases due to their regulation of diverse physiological processes and cell-surface accessibility (6, 71). Thus, unbiased and in-depth profiling of GPCR protein expression in a disease animal model or human clinical specimens would enable efficient discovery of potential regulators that drive disease phenotypes (22, 72, 73). Among the 99 DE GPCRs identified from brain regions of depression or anxiety model, 51 are previously reported to mediate depressive- or anxiety-like behaviors in animal models while the remaining ones have never been linked to these psychiatric disorders before. Of note, by integrating multiple informatics workflows to analyze two mental disease models established by disparate procedures, our study significantly expanded the mental stress-perturbed GPCR proteome by reporting 38 new potential GPCR regulators not overlapping with our previous study (22). The impressive efficiency of our TM proteomic analysis for screening potential drug targets is further illustrated by discovering the rapid antidepressant effect of an unreported GPCR ligand, prosaptide TX14(A). Prosaptide TX14(A) is an active fragment of the secreted neuroprotective and glioprotective factor prosaposin (74), and is able to cross the blood–brain barrier through a nonspecific mechanism (75). It has been reported to have the potential therapeutic values in the treatment of neurological disorders such as Parkinson`s disease (74), neuropathy, and neuropathic pain (76, 77). Prosaptide is an agonist for both GPR37L1 and GPR37, yet only GPR37L1 was found in our study to be downregulated in brain regions of the depression model (Fig. 3*C*). Prosaptide-mediated GPR37L1 signaling exhibits neuroprotective actions possibly by reducing N-methyl-D-aspartate receptor activity and influencing the release and reuptake of neurotransmitters such as dopamine and glutamate (66, 74), which may partially explain its antidepressant activity. Our study implicates an unappreciated role of prosaptide in reversing depressive-like behaviors, prompting further investigation into its molecular mechanism and the real GPCR target *in vivo*.

In summary, we expect further mining of the data resource and utilization of the brain TM protein spectral library provided by our study would catalyze new discoveries and deepen our understanding of brain physiology and disease mechanisms.

## Data Availability

The DDA/DIA MS raw data and spectral libraries generated during this study are available in the ProteomeXchange Consortium *via* the iProX partner repository (78, 79) with the dataset identifiers PXD027559 (URL: https://proteomecentral.proteomexchange.org/cgi/GetDataset?ID=PXD027559) and PXD047251 (URL: https://proteomecentral.proteomexchange.org/cgi/GetDataset?ID=PXD047251). The search result files of DIA-NN (.tsv) and Spectronaut (.sne) are available with the dataset identifier PXD047251. All annotated spectra could be visualized using the.sne files generated by Spectronaut. In addition, AlphaMap (https://github.com/MannLabs/alphamap) allows for visualizing spectra for individual peptides from each protein identified by DIA-NN.

## Supplemental Data

This article contains supplemental data.

## Conflict of interest

The authors declare that they have no competing interests.
