# Supplementary material for: Generation of a Deep Mouse Brain Spectral Library for Transmembrane Proteome Profiling in Mental Disease Models
Source: Mol Cell Proteomics. 2024 Apr 25;23(6):100777. doi: 10.1016/j.mcpro.2024.100777 (PMC11137342; doi:10.1016/j.mcpro.2024.100777)
Supplement: Supplemental Data [file mmc1.pdf]

Generation of a deep mouse brain spectral library for transmembrane proteome profiling in mental disease models

Shanshan Li[1,2#], Huoqing Luo[3,4,5#], Pan Tang[2,3,4], Cuiping Tian[2], Ji Hu[3,6*], Haojie Lu[1*], Wenqing Shui[2,3*]

[1]Institutes of Biomedical Sciences and Department of Chemistry, Fudan University, Shanghai, 200032, China.

[2]iHuman Institute, ShanghaiTech University, Shanghai 201210, China

[3]School of Life Science and Technology, ShanghaiTech University, Shanghai 201210, China

[4]University of Chinese Academy of Sciences, Beijing 100049, China

[5]Department of Anesthesiology & Perioperative Medicine, Xijing Hospital, Fourth Military Medical University, Xi'an 710032, China

[6]CAS Center for Excellence in Brain Science and Intelligence Technology, Chinese Academy of Sciences, Shanghai 200031, China

[#]Equal contribution

*To whom correspondence should be addressed to:

Wenqing Shui Email: shuiwq@shanghaitech.edu.cn

Haojie Lu Email: luhaojie@fudan.edu.cn

Ji Hu Email: huj@shanghaitech.edu.cn

Legends for Supplemental Figures

Figure S1. Comparison of three spectral libraries

Figure S2. Proteome identification and quantification performance in the depression model data analysis with two library-free workflows

Figure S3. Comparison of DE TM proteins quantified in three regions

Figure S4. Comparison of DE TM proteome in depression model vs anxiety model

Figure S5. Comparison of TM proteome regulation in depression model vs anxiety model analyzed by the SN-HB workflow

Figure S6. Behavior tests of prosaptide TX14(A) in naïve mice

Figure S7. Behavior tests of mice treated by ketamine or prosaptide TX14(A)

Legends for Supplemental Tables

Table S1. Protein quantity, peptide numbers and protein coverage in the depression model with three library-based workflows

Table S2. Protein quantity, peptide numbers and protein coverage in the anxiety model with three library-based workflows

Table S3. DE TM proteins identified in the depression or anxiety model with three library-based workflows

Table S4. References for disclosed regulators of depression or anxiety

Table S5. Variable DIA windows

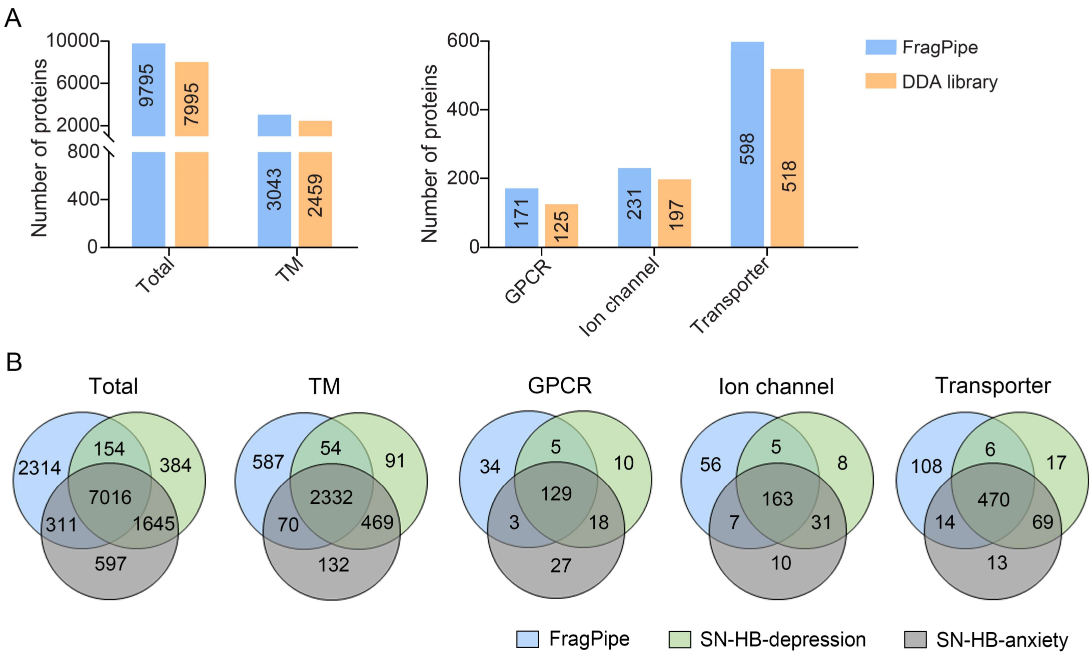

Fig. S1. Comparison of three spectral libraries. *A*, Number of proteins from FragPipe library and the DDA library. DDA library is from our previous work. *B*, Venn plots of total proteins, TM proteins, GPCRs, ion channels and transporters from our three libraries.

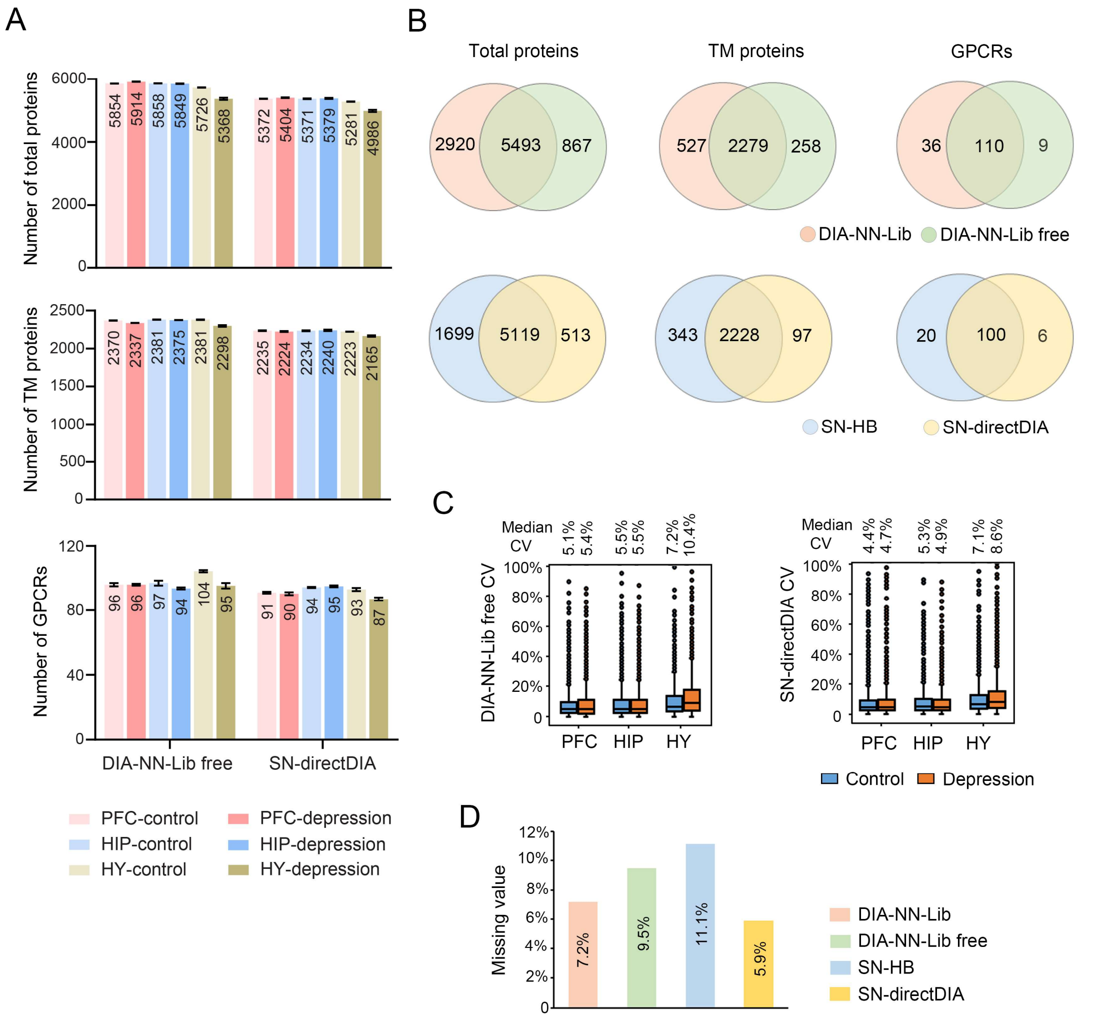

Fig. S2. Proteome identification and quantification performance in the depression model data analysis with two library-free workflows. *A,* Average number of protein IDs in each brain region with two analysis workflows (DIA-NN-Lib free, DIA-NN with library-free mode; SN-directDIA, Spectronaut with directDIA mode). *B,* Comparison of the total proteins, TM proteins or GPCRs reported by two analysis workflows. *C,* CV distribution of TM proteins from specific brain regions, indicating the high reproducibility of two library-free workflows. *D,* Missing values for both library-based and library-free workflows.

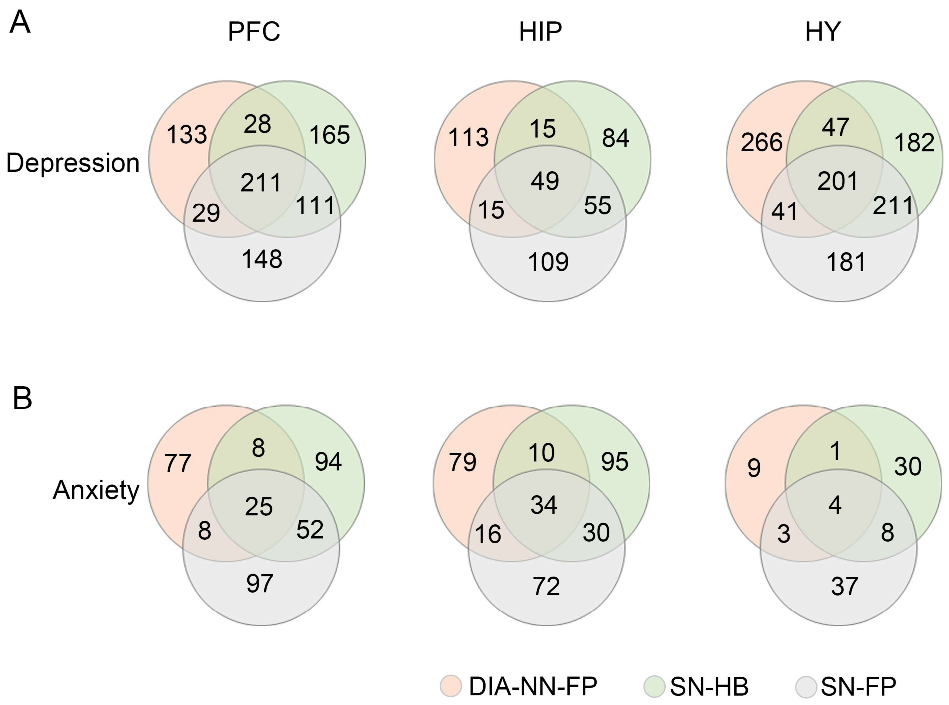

Fig. S3. Comparison of DE TM proteins quantified in three regions. Venn plots of DE TM proteins discovered by three workflows in depression model (*A*) and anxiety model (*B*).

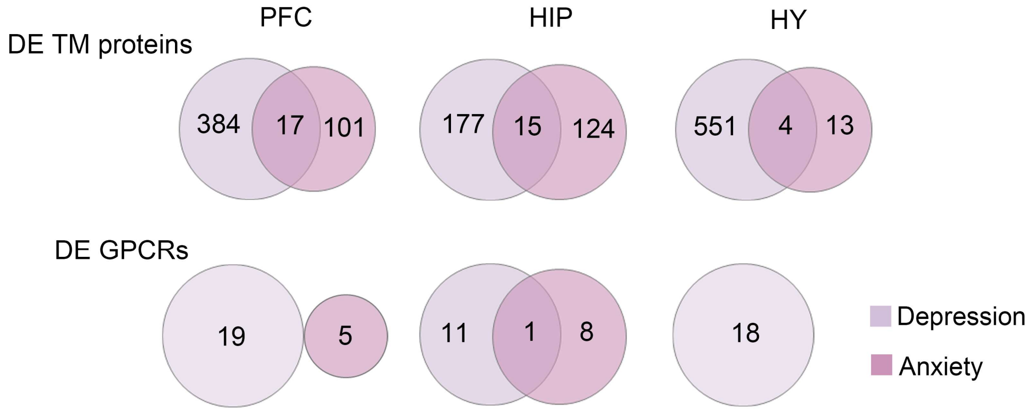

Fig. S4. Comparison of DE TM proteome in depression model *vs* anxiety model. Venn plots of DE TM and GPCR proteins in three regions of two models.

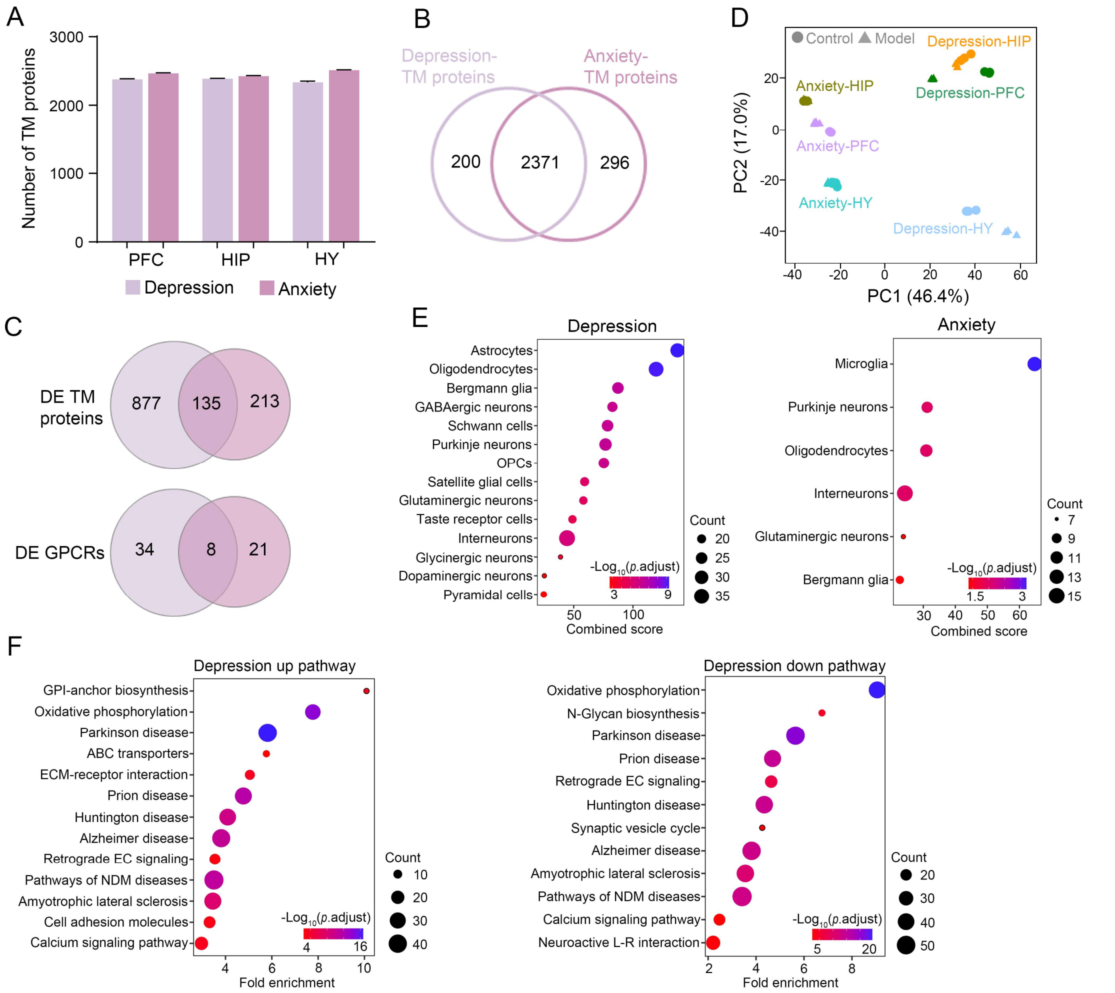

Fig. S5. Comparison of TM proteome regulation in depression model *vs* anxiety model analyzed by the SN-HB workflow. *A,* Number of total TM proteins quantified in three regions of depression and anxiety models. *B,* Venn plots showing the overlap of the TM proteins profiled in two mouse models. *C,* Venn plots of DE TM or GPCR proteins from three regions of two models. *D,* PCA plot of three regional expression of total TM proteins demonstrates the clustering of control, depression or anxiety groups, with distinct separation of two models. *E*, Significantly enriched cell types (adjusted *p* <0.05) of DE TM proteins from two models. *F,* Significantly enriched pathways (adjusted *p* <0.01) of up- or down-regulated TM proteins from the depression model. No significant enriched pathways are obtained for anxiety model.

OPCs, Oligodendrocyte Progenitor Cells; Retrograde EC signaling, Retrograde endocannabinoid signaling; Pathways of NDM diseases, Pathways of neurodegeneration − multiple diseases

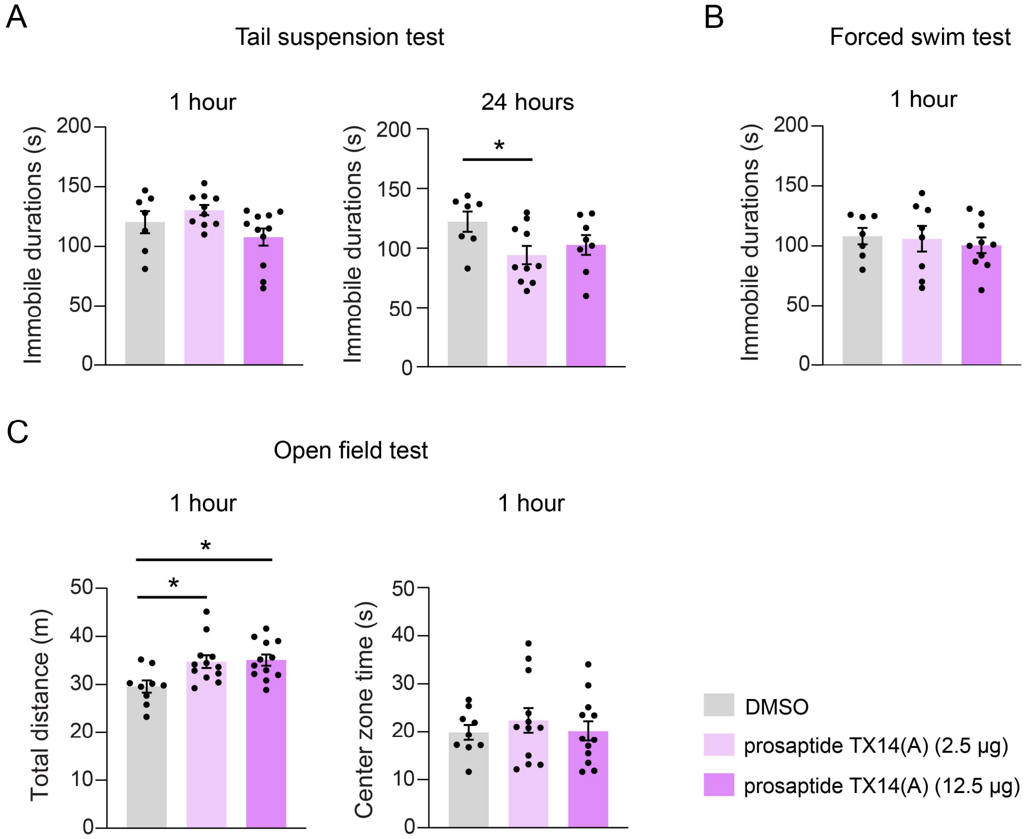

Fig. S6. Behavior tests of prosaptide TX14(A) in naïve mice. Antidepressant effects of stereotactic infusion of prosaptide TX14(A) (1.25 and 6.25 µg each side) into the mPFC of naïve mice at 1 and 24 hours after infusion as measured in TST (*A*), FST (*B*) and OFT (*C*). Data are mean ± SEM, **p* <0.05.

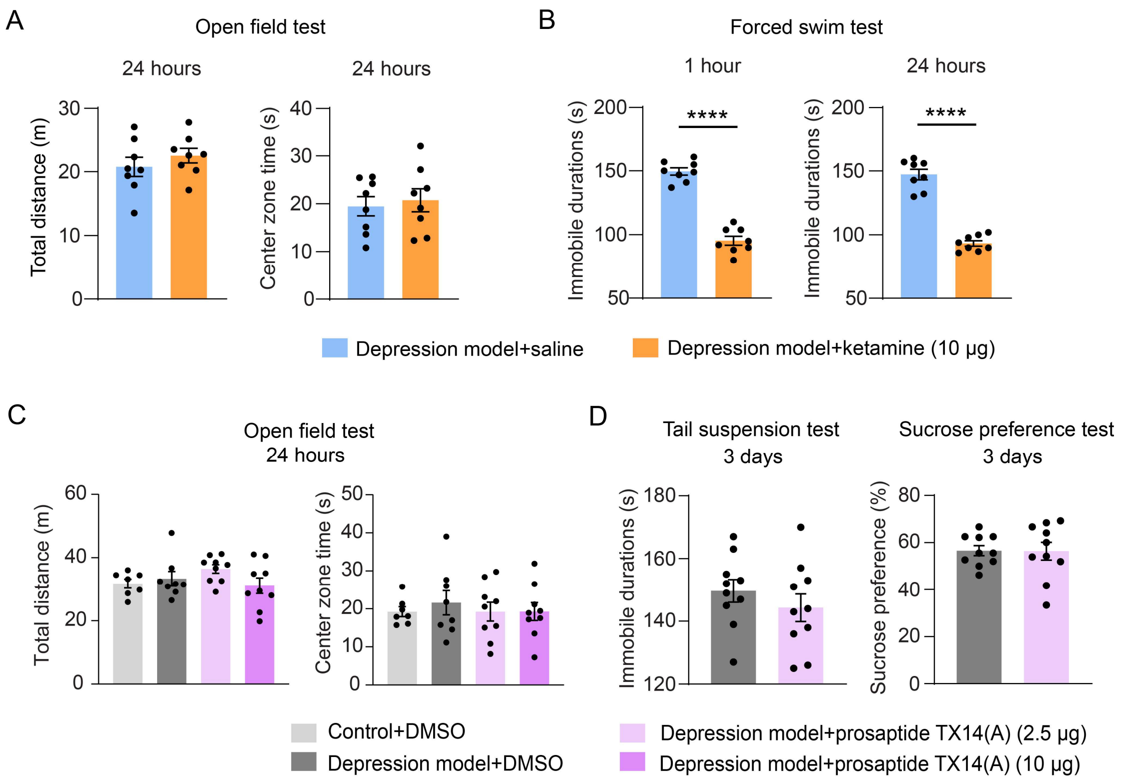

Fig. S7. Behavior tests of mice treated by ketamine or prosaptide TX14(A). *A,* No effect was observed for ketamine at 24 h post-infusion as measured in OFT. *B,* Antidepressant effects of stereotactic infusion of ketamine at 1 hour and 24 hours post-infusion as measured in FST. *C, D,* No antidepressant effects of stereotactic infusion of prosaptide TX14(A) (1.25 and 5 µg each side) into the mPFC at 24 h *(C)* or 3 days *(D)* post-infusion as measured in the OFT *(C)*, TST *(D)* and SPT *(D)*.

Data are mean ± SEM, ****p <0.0001.
